# Dual Intrauterine Device Placement for Refractory Endometriosis Pain in a Bicornuate Uterus: A Case Report

**DOI:** 10.7759/cureus.114635

**Published:** 2026-08-17

**Authors:** Mohammad Mustafa Farooq Khan, Saadia Ejaz

**Affiliations:** 1 Department of Obstetrics and Gynaecology, Sligo University Hospital, Sligo, IRL

**Keywords:** bicornuate uterus, dysmenorrhea, endometriosis, hysteroscopy, levonorgestrel, pelvic pain

## Abstract

A 27-year-old nulliparous woman presented with chronic pelvic pain (CPP) (8/10 on the Numerical Rating Scale (NRS)), dysmenorrhea, heavy menstrual bleeding (HMB), and a family history of endometriosis. She had failed treatment with an etonogestrel implant and dienogest. Hysteroscopy confirmed a bicornuate uterus. Under direct hysteroscopic guidance, two levonorgestrel-releasing intrauterine systems (LNG-IUS) were inserted, with one device placed in each uterine horn. At the four-month follow-up, the patient reported a significant reduction in pain (2/10 on the NRS), dysmenorrhea, and bleeding, with ultrasound confirming correct device placement. At six months, her pain score had decreased to 1/10. Dual LNG-IUS insertion under hysteroscopic guidance represents a minimally invasive and effective strategy for managing endometriosis-related pain in patients with a bicornuate uterus when conventional therapies are inadequate. Long-term follow-up and regular monitoring are warranted to assess for potential side effects.

## Introduction

Chronic pelvic pain (CPP) is defined as constant, dull, aching pain, particularly in the stomach or pelvic region, which can persist for up to six months irrespective of any menstrual, intercourse, or pregnancy-associated complication [1]. Globally, CPP affects approximately 15% of women in the general population and 25% of women of reproductive age, making it one of the most common complaints among women presenting to outpatient gynecology departments [2]. Risk factors and associated symptoms of CPP include dysmenorrhea, dyspareunia, dysuria, and dyschezia [1]. Endometriosis is a leading cause of CPP and subfertility, affecting more than 10% of women of reproductive age [3]. While laparoscopy remains the gold standard for definitive diagnosis, clinical diagnosis based on characteristic symptoms often serves as the initial step in management.

The management of CPP often begins with hormonal suppression as a first-line strategy, which reduces pain by promoting atrophy of endometriotic lesions through suppression of ovarian activity. Commonly prescribed treatments include combined oral contraceptive pills (COCPs), progestogens (oral or implantable formulations, including intrauterine devices (IUDs)), and gonadotropin-releasing hormone (GnRH) agonists and antagonists. These treatment options have demonstrated effectiveness in reducing dysmenorrhea and non-menstrual pelvic pain [4]. Another effective treatment option is the levonorgestrel-releasing intrauterine system (LNG-IUS). The 19-nortestosterone derivative progestin, levonorgestrel, induces atrophy of endometrial glands and decidualization of stroma, thereby creating an estrogen-deficient environment. LNG-induced immediate suppression of the endometrium is essential in lowering menstrual bleeding and endometriosis-induced pain [5].

A rare but significant challenge faced by gynecologists is the presence of Müllerian anomalies, including a bicornuate uterus. With an overall incidence of approximately 0.4%, this defect results from incomplete fusion of the Müllerian ducts between the sixth and 10th weeks of gestation during embryogenesis [6]. According to the American Society for Reproductive Medicine (ASRM) Müllerian Anomalies Classification 2021 (MAC2021), Müllerian anomalies are now categorized into nine groups based on anatomical features, replacing the historical numbered classification system. Under the new classification, a bicornuate uterus is categorized as a “Dysfusion Anomaly” and is defined by an external fundal indentation of >1.0 cm [7]. A bicornuate uterus has a unique anatomy, characterized by a heart-shaped external contour and two distinct uterine cavities [8]. It is further classified according to the number of cervices present: bicornuate unicollis has a single cervix, whereas bicornuate bicollis comprises two separate spaces with their respective cervices [9].

The unique anatomy of a bicornuate uterus poses challenges for patients using an IUD, with an increased risk of expulsion, pregnancy, bleeding, perforation, and pain [10]. The limited efficacy of a single IUD placed in one uterine horn is further compounded by delayed diagnosis of the anomaly, as it often remains asymptomatic for most of its course, which can still allow for conception in the other horn of the uterus [11]. These challenges are further exacerbated by the limited efficacy of hormonal therapies. Estrogen dependency and progesterone resistance are key factors that may not only limit treatment efficacy but also contribute to symptoms and comorbidities, including but not limited to autoimmune disorders, inflammatory pathways, and mental health conditions [12].

This case report describes a patient with a bicornuate uterus who presented with heavy bleeding and severe pain that was refractory to conventional therapeutic interventions, raising clinical suspicion of clinically diagnosed endometriosis. The novelty of this report lies in its description of a hysteroscopy-guided dual LNG-IUS placement for the management of refractory pain in a bicornuate uterus, an approach scarcely reported in the literature. This strategy allowed the entire endometrium to be equally supplemented by progestin therapy, overcoming the limitation imposed by the anatomical anomaly. Furthermore, this report demonstrates successful resolution of the patient’s symptoms and the feasibility of this surgical intervention, underscoring its potential to provide relief to a complex patient population with limited treatment options.

## Case presentation

A nulliparous female in her late 20s presented to our Gynecology Outpatients Department with the chief complaint of intractable CPP and was referred by her general practitioner (GP). The patient described a constant, dull pain that had been present since menarche at the age of 14 and was not relieved by analgesics. The pain was not related to her dietary habits. The patient also reported severe dysmenorrhea, heavy menstrual bleeding (HMB), and intermenstrual spotting. As the patient was sexually active, she also described dyspareunia and post-coital bleeding. Given these symptoms, her GP had previously inserted an etonogestrel contraceptive implant (Implanon), which did not provide symptomatic relief. The patient rated her baseline pain as 8/10 on the Numerical Rating Scale (NRS).

The patient’s medical history indicated that she was a non-smoker and non-drinker, with a height of 1.67 m, a weight of 98.6 kg, and a BMI of 35.4 kg/m². Her past medical and surgical history included acne, hirsutism, gastroenteritis treated with fluoxetine and esomeprazole, knee surgery, and tonsillectomy. Her family history was significant for endometriosis in her mother, as well as ischemic heart disease and hypertension. Her key symptoms of early menarche, dysmenorrhea, dyspareunia, and HMB, together with a positive family history, suggested an increased risk of endometriosis. This underscores the importance of a detailed medical history in informing diagnostic reasoning. The subsequent confirmation of a bicornuate uterus presented a management challenge, as standard treatment regimens are often suboptimal in the presence of anomalous anatomy. The patient expressed a desire for future fertility; therefore, the management plan was designed to address both her pain and her reproductive goals.

Timeline of presentation and management

**Figure 1:**
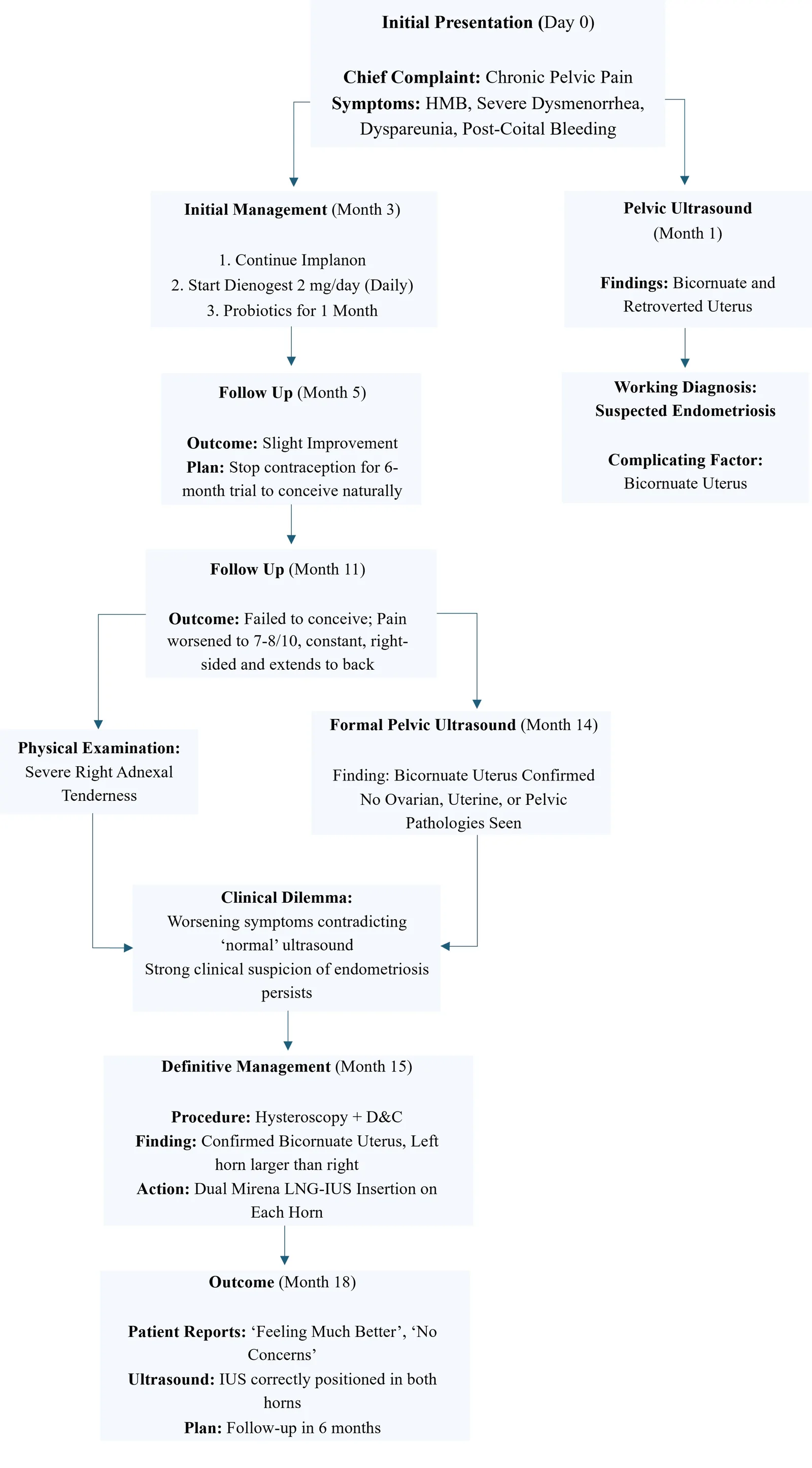
Timeline of presentation and management HMB: heavy menstrual bleeding; D&C: dilation and curettage; LNG-IUS: levonorgestrel-releasing intrauterine system Chart created by the authors

Figure 1 illustrates the timeline of the patient's presentation to the resolution of her chief complaint.

Investigations

Baseline Investigations

Initially, a pelvic ultrasound was performed to assess the uterine anatomy. The scan reported findings consistent with a retroverted bicornuate uterus. The ultrasound was otherwise reported as “normal,” with no other pathology identified. This anatomical finding was critical in guiding subsequent management decisions.

Crucial Investigations

The patient returned several months later with worsening, excruciating pain, scoring 9/10 on the NRS, localized to the right lower abdomen and radiating sharply to the back. A repeat pelvic ultrasound later that year again reported a bicornuate, retroverted uterus, with no ovarian, uterine, or other pelvic pathology identified. Although ultrasound is excellent for visualizing uterine anatomy, it has limited sensitivity for detecting endometriosis. Clinical examination at this time revealed right adnexal tenderness, which, in the context of her symptoms, increased the clinical suspicion for endometriosis despite the normal ultrasound findings.

It is important to acknowledge a limitation of the diagnostic pathway. While the ultrasound was adequate for identifying a bicornuate uterus, no MRI or laparoscopy was performed to definitively distinguish a bicornuate uterus from a septate uterus or to confirm endometriosis visually. Diagnostic laparoscopy was discussed with the patient. In accordance with National Health Service Executive (HSE) guidelines, which recommend first-line hormonal management for endometriosis-associated pain [13], and considering the patient’s preference to trial effective medical therapy, the decision was made to proceed with therapeutic intervention. The patient remained on a waiting list for diagnostic laparoscopy as part of her potential future management.

The definitive diagnostic procedure was hysteroscopy, which confirmed a bicornuate uterus with a distinct septum dividing the two cavities. The left horn of the endometrial cavity appeared significantly larger than the right horn. These findings confirmed the anatomical basis for the planned intervention.

Differential diagnosis

Endometriosis was the principal diagnosis throughout the course of the patient’s management (Table 1). To exclude other potential causes and complications, a pelvic ultrasound was performed, which revealed no ovarian pathology. Gastrointestinal or urological causes were considered but were considered less likely, as the patient reported no association between her pain and dietary or urinary habits. Other conditions, such as chronic pelvic inflammatory disease (PID) or adenomyosis, were also considered less likely, as her reported symptoms and examination findings did not align with their classic presentations, and the ultrasound did not show typical features. In addition to imaging, the patient’s strong family history provided further supporting evidence. The final clinical diagnosis was endometriosis, complicated by the presence of a bicornuate uterus.

**Table 1:** Differential diagnosis and rationale for clinical decision-making This table summarizes the diagnostic reasoning process

Working diagnosis	Supporting evidence	Rationale for exclusion
Endometriosis	Chronic pelvic pain since menarche, severe dysmenorrhea, dyspareunia, heavy menstrual bleeding, and a positive family history	Remained the principal diagnosis throughout management
Ovarian pathologies	Right-sided abdominal pain and tenderness on examination	Formally ruled out by pelvic ultrasound (month 14), which reported no ovarian pathologies
Chronic pelvic inflammatory disease (PID)	Chronic nature of pelvic pain	Absence of classic symptoms (e.g., fever, discharge); clinical presentation and history were not aligned
Adenomyosis	Heavy menstrual bleeding and dysmenorrhea	Reported symptoms and examination findings did not fully align with the classical presentation; ultrasound did not show typical features
Gastrointestinal or urological causes	Non-specific chronic pelvic pain	Patient reported no linkage between pain and her dietary or urinary habits

Treatment

Initial Pharmacological Management (Day 0)

After the initial pelvic ultrasound, clinicians suspected endometriosis, and the patient was started on a trial of dienogest (2 mg/day), while her existing etonogestrel implant (Implanon) was continued. The primary goal was to achieve pain relief, despite the patient’s desire for a future pregnancy. She was also advised to take probiotics for one month. At the month-four follow-up, the patient reported some improvement in pain but reiterated her strong desire for fertility. A six-month period of attempting conception was agreed upon before considering surgical management. However, after six months without conception (at month 11), her pain severity increased to 9/10 on the NRS, prompting a shift to surgical management.

Surgical Intervention (Month 14)

The patient provided consent and was admitted for hysteroscopy with possible dual LNG-IUS insertion under general anesthesia. The procedure was performed using a 5-mm Karl Storz Bettocchi hysteroscope with a 5-Fr working channel (Karl Storz, Tuttlingen, Germany).

**Figure 2:**
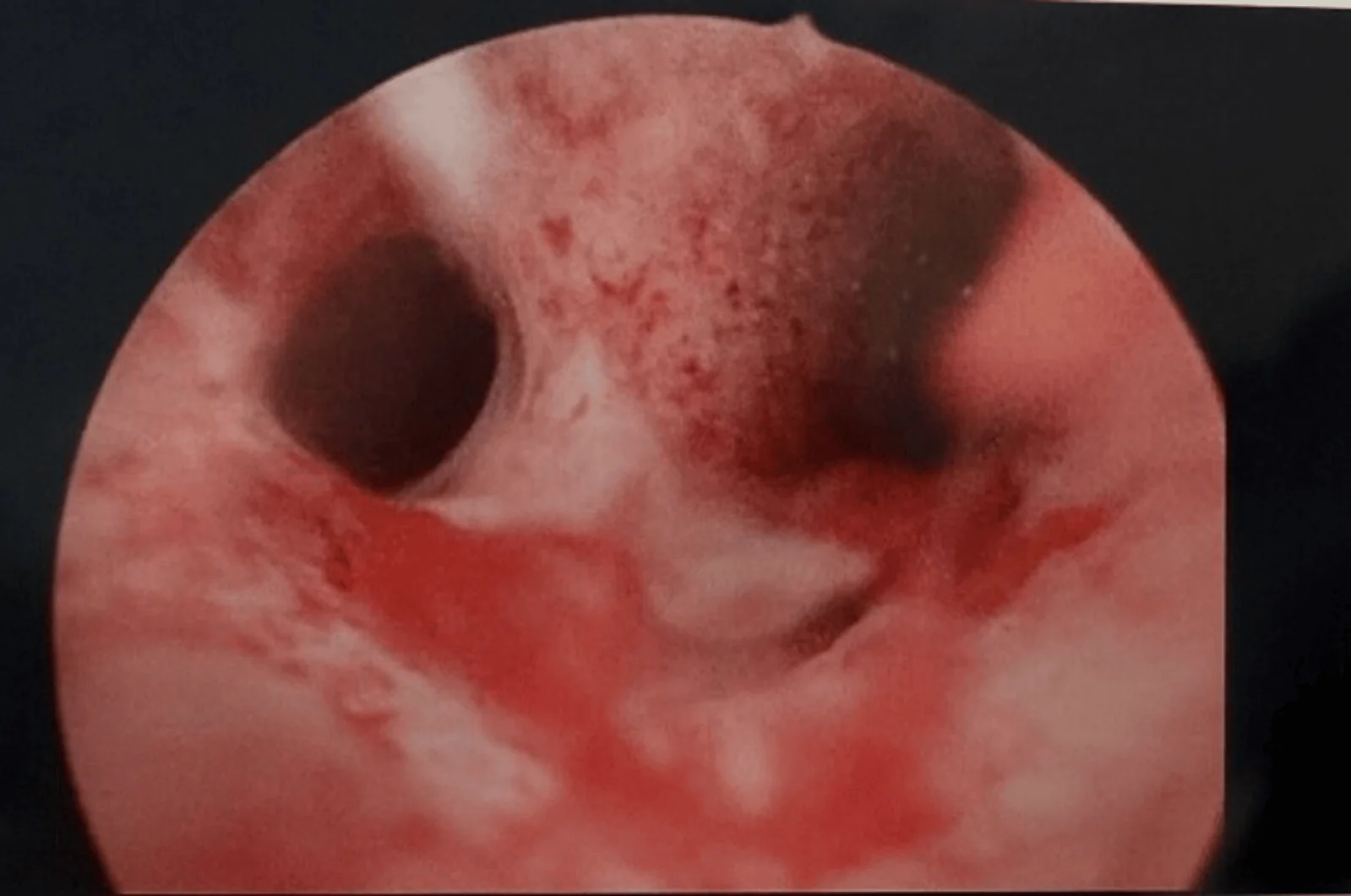
Hysteroscopy showing no orbital uterus, two cavities (bicornuate)

**Figure 3:**
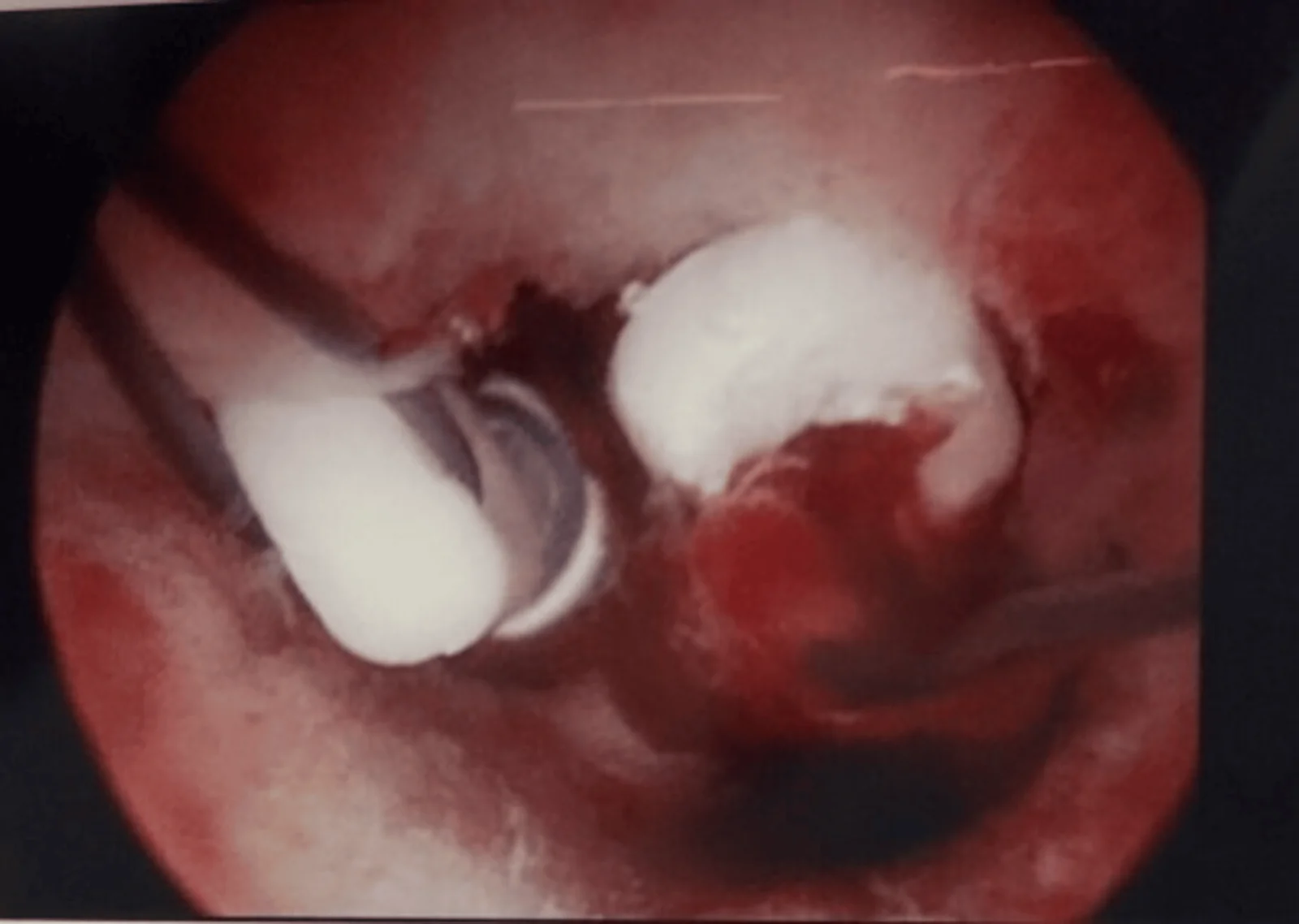
Hysteroscopy: post-insertion of two Mirena coils, one in each cavity

Under aseptic conditions, with the patient in the lithotomy position, the cervix was visualized and dilated to Hegar size 6. Hysteroscopy confirmed a bicornuate uterus with a distinct septum and a larger left horn (Figure 2). Under direct hysteroscopic vision, two Mirena (LNG-IUS) devices were inserted sequentially. Each IUS was loaded into its standard introducer, which was then passed through the hysteroscope’s working channel. This enabled sequential, visually guided deployment of one IUS into the fundal region of each uterine horn. Correct positioning was confirmed hysteroscopically at the time of the procedure (Figure 3).

The rationale for dual LNG-IUS placement was to ensure adequate levonorgestrel delivery to the entire endometrium. A single IUD in one horn is often ineffective and carries a risk of pregnancy in the contralateral horn.

Outcome and follow-up

**Figure 4:**
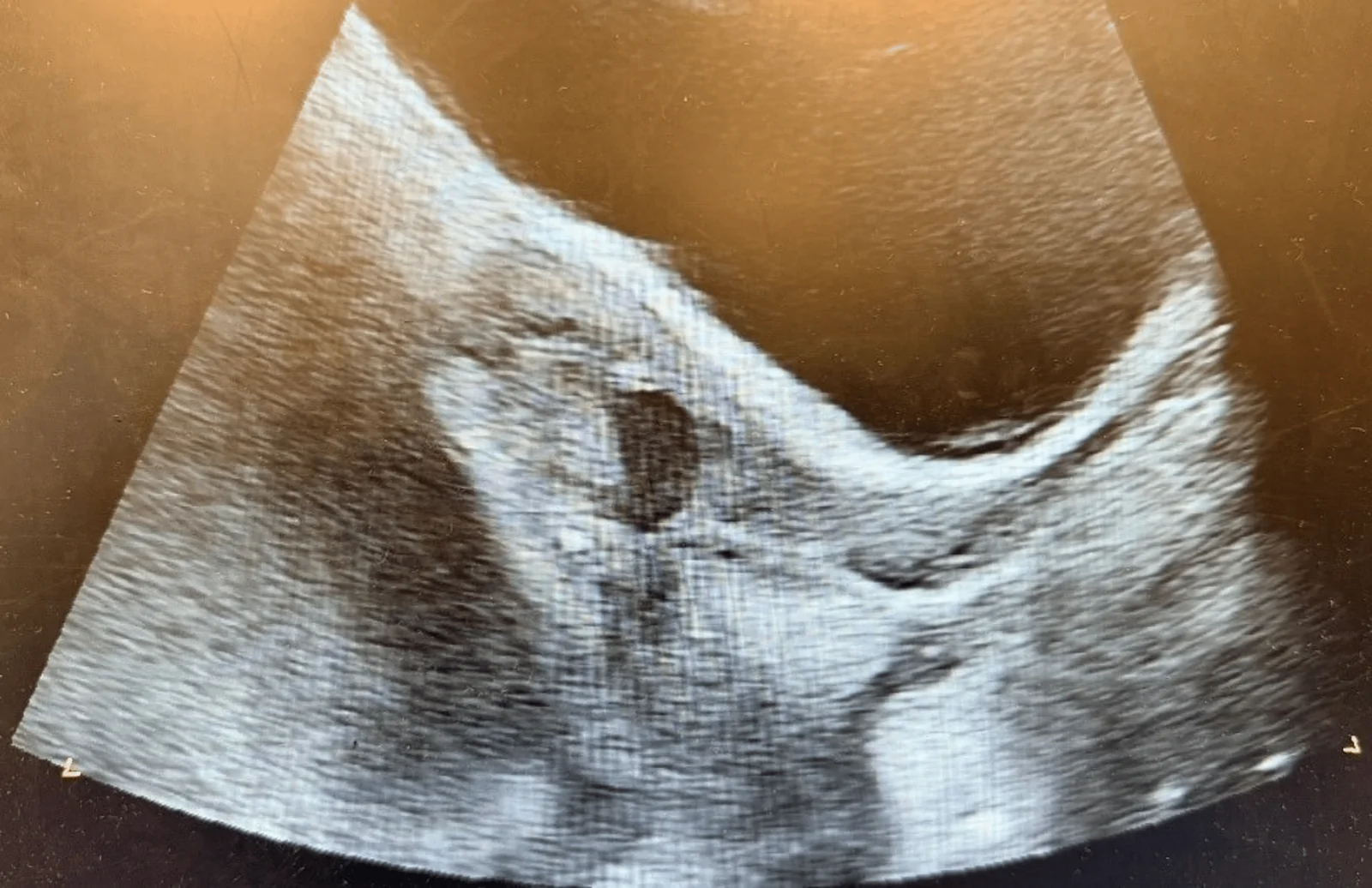
Pelvic ultrasound showing bicornuate uterus with IUCDs in situ No ovarian or pelvic pathology is observed IUCDs: intrauterine contraceptive devices

Post-procedure, the patient remained stable and was discharged with analgesics. She was advised to return for follow-up after four months (month 18). At that visit, she reported significant clinical improvement, with pain scores reduced to 2/10 on the NRS and marked reductions in dysmenorrhea and HMB. Objective confirmation was obtained via transabdominal and transvaginal pelvic ultrasound. The report confirmed a bicornuate uterus, with an appropriately positioned IUCD within each uterine horn. Both ovaries appeared normal, and no free fluid was noted (Figure 4).

The patient's health status improved significantly, with further reduction in pain. At a subsequent six-month follow-up (month 20), her pain score was 1/10 on the NRS. She was scheduled for continued long-term follow-up to monitor treatment efficacy and potential adverse effects. Given her continued desire for future fertility, future management will involve discussion of IUS removal to support her reproductive goals.

## Discussion

This case report demonstrates the successful management of refractory pelvic pain, clinically attributed to endometriosis, in a patient with a bicornuate uterus using hysteroscopy-guided dual LNG-IUS insertion. This approach resulted in a significant reduction in pain, from a baseline of 8/10 to 1/10 on the NRS at six months postoperatively. A significant challenge was the presence of a bicornuate uterus. The WHO identifies such anomalies as Category 4 for IUD use, indicating a setting in which the risks of malposition, expulsion, and uterine perforation may outweigh the benefits [14]. Furthermore, a single IUD placed in one horn is often ineffective for symptom control and carries the risk of unintended pregnancy in the contralateral horn, which, in the majority of cases, may result in miscarriage or pregnancy termination [15]. Our patient had also failed systemic progestin therapy, a common first-line treatment for endometriosis-related pain [16,17].

The rationale for the dual-IUS approach was to ensure therapeutic delivery of levonorgestrel across the entire endometrial surface. While the literature on this specific application is scarce, the principle is supported by case reports describing dual IUD placement for contraception or bleeding management in other Müllerian anomalies, such as uterus didelphys [18]. However, its reported use for endometriosis-related pain in a bicornuate uterus is, to our knowledge, novel and has not been addressed in current systematic reviews. This underscores the need for tailored approaches when standard therapy falls short in complex anatomical settings.

A definitive laparoscopic diagnosis of endometriosis was deferred in favor of immediate therapeutic management. This clinical decision aligns with contemporary guideline-based practice [13], which prioritizes empirical hormonal treatment for symptom relief in suspected endometriosis. The patient's preference for a trial of effective medical management over immediate diagnostic surgery further supported this pathway. The subsequent resolution of symptoms with the LNG-IUS, a first-line treatment for endometriosis-associated pain, provides indirect support for the initial clinical diagnosis. We acknowledge that laparoscopy remains the diagnostic gold standard and remains part of the patient’s future care plan.

The technical success of hysteroscopy-guided placement underscores the value of direct visualization for precise IUD deployment in complex anatomy. However, this remains an off-label application with unknown long-term risks, particularly regarding device stability and future fertility outcomes following removal. Consequently, vigilant long-term follow-up is essential. This case underscores the need for more robust clinical evidence to guide the management of endometriosis in patients with concurrent Müllerian anomalies.

Learning points

For patients with refractory endometriosis symptoms and a complex Müllerian anomaly such as a bicornuate uterus, dual LNG-IUS insertion under hysteroscopic guidance represents a viable and effective therapeutic alternative when standard management has failed. Although this approach is off-label, it can provide significant symptomatic relief in anatomically challenging cases. However, its success hinges on maintaining a high index of suspicion for endometriosis in patients with uterine anomalies and classic symptoms, even when initial imaging studies are unrevealing, as atypical pelvic anatomy can easily mask or obscure the diagnosis. Before proceeding with such an intervention, thorough patient counseling is paramount. Clinicians must clearly discuss the potential risks and benefits while also acknowledging the limited robust long-term evidence supporting this dual-device strategy in patients with bicornuate uteri. Equally important is a commitment to long-term follow-up, which serves multiple critical purposes, including monitoring for device expulsion, managing any emerging side effects, and, most importantly, facilitating ongoing discussions about future fertility planning, given the unique interplay between the patient's uterine anatomy and the presence of intrauterine devices.

## Conclusions

This case report describes the successful management of clinically diagnosed endometriosis in a patient with a bicornuate uterus using a dual LNG-IUS system. Hysteroscopically guided placement facilitated uniform endometrial suppression, leading to complete resolution of pain and associated menstrual symptoms where single-IUD and systemic therapies had failed. This underscores the potential of tailored, minimally invasive approaches in anatomically complex cases. Based on this experience, we recommend that clinicians engage in detailed, informed discussions with patients when proposing off-label interventions. Future research should focus on the long-term efficacy, expulsion rates, and fertility outcomes associated with dual LNG-IUS use in anomalous uteri, as well as comparative studies evaluating this approach against standard therapeutic options.
